# Toward a Unification of the Arts

**DOI:** 10.3389/fpsyg.2018.01938

**Published:** 2018-10-24

**Authors:** Steven Brown

**Affiliations:** Department of Psychology, Neuroscience & Behaviour, McMaster University, Hamilton, ON, Canada

**Keywords:** arts, production, perception, supervenience, multisensory

## Abstract

This article presents a manifesto for the scientific exploration of the arts in their totality, rather than conceiving of each artform independently on its own terms. In order to achieve this, I present an analytical procedure that is comprised of two related steps. The first step is to identify instances of sharing in the production mechanisms across artforms, for example the occurrence of rhythmic structure in music, dance, and poetry. The second is to examine how this sharing creates “affordances for combinations,” making it possible for music to be set to a poem or for dance movements to be choreographed to music. By elucidating the neurocognitive mechanisms of sharing across arts domains and the affordances that they offer for creating combinations, it should be possible to achieve a unification of the arts.

## A Unification of the Arts

What do the arts share and why? What are the underlying similarities and differences among the arts, both at the cognitive and neural levels? While each branch of the arts shows domain-specific features that define it as a distinct branch of the arts, artforms also show a strong capacity to be combined with one another to create syntheses, as seen in dancing to music, singing words, streaming background music in a movie (Gorbman, 1987; Cohen, 2013), or blending sounds and visual elements in multimedia forms (Tan et al., 2013; Costello, 2017). By understanding both the unique specializations of each artform and the manners in which artforms are able to combine to form syntheses, we can aspire toward a neurocognitive unification of the arts. Before presenting a road map for how to achieve this unification, I will start out with some general definitions of the arts as well as discuss historical notions of what the arts share and how artforms can be combined.

**FIGURE 1:**
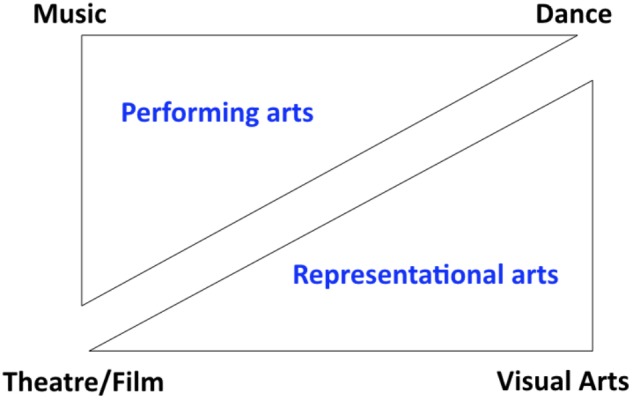
A classification of the arts. The figure shows the four major branches of the arts as making up a tetrad. Within that are opposing triangles. The top triangle is made up of the performing arts, while the bottom triangle is made up of the representational arts. Theater/film and dance sit in both categories.

Standard characterizations place the arts within the purview of “expressive behaviors,” arguing that such behaviors tend to be both creative and driven by aesthetic considerations. Many of these behaviors take the form of public performances (Bell, 2008) – such as music, dance, and theater – although the rise of mass-media technologies has resulted in a situation whereby most people experience these performances in a mediated fashion, rather than in a live manner. Other arts-related behaviors produce static artifacts, such as paintings, sculptures, photographs, and books. There are multiple ways to classify the arts. For the purposes of this article, I will think about the arts in terms of the standard conception of “branches” found in the humanities, with the four major branches being music, dance, theater^1^ (and film, but also including oral forms of storytelling and poetry), and the visual arts, as shown by the tetrad in Figure 1. There are additional arts that employ the chemical senses, such as gastronomy and perfumery, that are not shown in the figure. I will next divide the tetrad into two opposing triangles. The top triangle comprises the “performing arts,” and therefore excludes the visual arts as static objects, such as paintings and sculptures. The bottom triangle comprises what I will refer to as the “representational arts,” since they can be used in a narrative fashion to convey information about objects, people, and events. Music is excluded from this grouping since it is generally unable to convey information referentially in the way that a sentence or picture easily can. It is not critical for this scheme that all forms of dance or visual art be representational. What is important is that these artforms have the potential to be representational and that this is a critical feature that distinguishes them from music. Note that in the double-triangle representation, theater/film and dance sit in both categories. This classification reflects the fact that the arts, in general, serve two primary functions, namely the conveyance of narrative (theater, film, the visual arts, and narrative forms of dance) and the promotion of interpersonal coordination (dance and music). The narrative arts function to tell stories, often to support social learning through the modeling of prosocial behaviors (Boyd, 2009; Gottschall, 2012) and the stimulation of social cognition (Mar and Oatley, 2008), whereas the coordinative arts function to stimulate group participation, thereby serving as a symbol of group unity, a reinforcer of group affiliation, and a promoter of cooperation (Brown, 2000; Reddish et al., 2013; Launay et al., 2016). In fact, the promotion of social cooperation unites all of the arts from a functional perspective. Of course, there are solo forms of the performing arts as well, such as piano recitals, but even they serve a coordinative function by synchronizing the expression of emotion in audience members.

Two functions that are invariably mentioned in definitions of the arts – and the arts in relation to them – are aesthetics and creativity. In my opinion, these functions are the *least* specific features of the arts. I see both of them as being generic. The arts cover a far broader set of processes than aesthetics alone (Brown and Dissanayake, 2009), and likewise aesthetics applies quite generally to our sense of liking for objects, be they art objects or anything else (Brown et al., 2011). The same argument can be made about the generic nature of creativity, which is the process of generating novel ideas. Creativity can apply to the generation of art works, but it applies to the generation of any type of novel idea or product, be it an art work or anything else (Boden, 2004; Kozbelt et al., 2010; Ward and Kolomyts, 2010). Aesthetics and creativity are supervenient concepts that apply to *all* domains, not just the arts. As a result, they have no specificity for the arts.

I suggest instead that there are four other functions that truly are specific for the arts and that comprise genuine “art modules” at the neurocognitive level. While the focus of this article is on what the arts share, I will briefly describe this issue of art specificity for the sake of completeness. Two of the functions are narrative and two of them are coordinative. (1) *Figurative drawing*: generating figurative representations of objects, people, or events, but doing so in two dimensions rather than three (i.e., through flattening), mainly in the form of pictures. (2) *Acting*: performing a re-creation of a person by pretending to be that person through character portrayal. It occurs predominantly in theatrical works, although it also occurs to a lesser extent in storytelling and in narrative forms of dance, for example ballet. (3) *Metric entrainment*: interpersonal synchronization of movement or sounding that occurs in a regular, isometric rhythm. This process is shared between music and dance. (4) *Tonality*: the organization of musical pitch-space according to scaled pitches that are used recurrently in the generation of melodies and harmonies. It is specific to music. The neural implication of the claim that these four functions are art-specific modules is that there should be a certain degree of neural specificity for them in the human brain and even that these functions might be evolutionary novelties, not just behaviorally but neurally. Unfortunately, many of the relevant experiments have not yet been carried out. For example, there is no published work on the neural basis of dramatic acting or work comparing the processing of 2-dimensional and 3-dimensional visual-art objects. Much neuroimaging work on sensorimotor entrainment is polarized between models that implicate the basal ganglia and those that implicate the cerebellum (Chauvigné et al., 2014). Despite tonality being a domain-specific cognitive feature of music, no work thus far has provided compelling evidence for the localization of music distinct from that of speech processing (Peretz et al., 2015). Hence, much work is needed in order to advance the case for neural specificity of the arts. From this point on, I will focus on the process of sharing among the arts, rather than on distinction.

## The Arts Combined

We can conceive of the unification of the arts with regards to two key issues: what the arts share, and how the arts combine. The idea that the arts have intrinsic similarities and that they can be combined to create syntheses has historical roots in Enlightenment thinking. In the 18th century, a variety of treatises developed the notion of “sister arts,” whereby artforms were seen as having have deep kinship based on psychological similarities between them (discussed in Malek, 1974). According to these treatises, music and poetry are united by the vocal expression of emotion; painting and poetry are united by their ability to conjure up visual images; and painting and music are united by their use of colors (tone-colors in the case of music) and expressiveness. Regarding combinations of the arts, the notion of a *Gesamtkunstwerk* or “total work of art” was established by aesthetic philosophers and practitioners in the 19th century (Wagner, 1849/1993; Smith, 2007; Brown and Dissanayake, 2018). It represented a grand unification of the arts, encompassing theater, music, dance, and visual design, as applied to literary themes from mythology, folklore, history, and religion. Brown and Dissanayake (2018) proposed that, long before European aesthetic philosophers had devised the notion of a total work of art, religious ceremonies in indigenous cultures had for millennia been syntheses of the arts on a similar scale and of a similar scope to a *Gesamtkunstwerk*, arguing for the co-evolution of religion and the arts.

While the current article focuses on between-domain syntheses, the more common means of discussing syntheses in the arts is at the within-domain level. It is quite common to think of artforms as being comprised of syntheses of constituent dimensions. Prominent examples include: (1) plot and character in the narrative arts; (2) narration and dialog in literature; (3) narration (diegesis) and character portrayal (mimesis) in oral storytelling and theater; (4) the duality between one’s self and the character that one is portraying during acting; (5) pitch and rhythm in music; (6) melody (the horizontal dimension) and harmony (the vertical dimension) in musical pitch; (7) movement patterns and rhythm in dance; (8) form, color, spatial organization, texture, implied depth, and implied movement in paintings; (9) dynamic and static components in multimedia artforms; and (10) taste and smell for flavor processing in chemical arts like gastronomy and enology.

**FIGURE 2:**
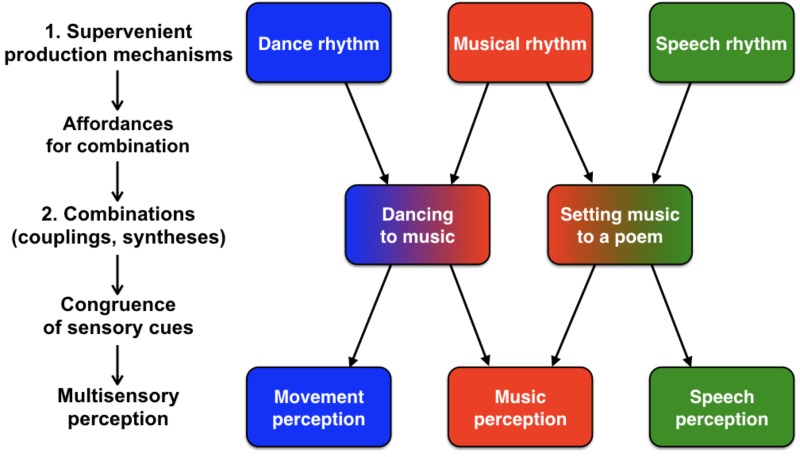
How artforms can combine. Shared production mechanisms between two or more artforms create affordances for combination, permitting a natural coupling between them in time and/or space. The combination of elements from these syntheses activates sensory processes for multiple systems in parallel. This often works out at the production level such that the elements from each artform are congruent and mutually reinforcing.

However, the focus of this article is on *between-*domain syntheses. In order to develop an understanding of such syntheses, I will argue that we need to consider two interrelated levels of analysis, as shown in Figure 2. The first step is to identify *shared production mechanisms* between two or more artforms. The example presented in the figure considers rhythm production to be a supervenient process shared by the production mechanisms of dance, music, and poetry. Importantly, this sharing of production-processes establishes *affordances for combination* that can be used by creators of artworks, permitting a natural coupling between artforms in time and/or space. The second step is to identify how such affordances allow creators to produce *combinations* (couplings) between two or more artforms to generate syntheses, as exemplified in the figure by choreographing dance to music or setting music to a poem, in both cases striving for an alignment of stress patterns in the respective metrical structures of dance, music, and speech. As will be discussed below in the section “Multisensory perception,” the combination of elements from these syntheses has an impact on perceivers of artworks since it activates sensory processes for multiple systems in parallel. This is often supported by creative processes occurring at the production level such that the elements from each artform are congruent and mutually reinforcing. For example, when choreographing dance to music, the choreographer will generally call for large dance movements to be associated with the strong beats (rather than the weak beats) in the music. Hence, the audience member’s perception of this dance/music combination integrates a large degree of optic flow from the dance with a large degree of acoustic energy from the music, resulting in a multisensory interpretation of intensity and strength.

### Supervenience in Production Mechanisms

I will now explore further examples of supervenience in the production-mechanisms of the arts. Narrative ideas can be conveyed using the “narrative triad” of language, pantomime, and drawing (Yuan et al., 2018). In other words, the same narrative idea can be depicted through speech, gesture, and graphic images. The conveyance of narrative through language can itself occur using the multiple motor modalities of speech, writing, and signing (among others). This sensorimotor diversity reflects the idea of “motor equivalence” (Kelso et al., 1998), whereby different effector pathways can generate the same or equivalent output. Similarly, a musical melody can be generated through vocalization at the larynx or by using the vocal tract in a non-phonatory manner to whistle or to blow air into an aerophone instrument like a flute. Likewise, a melody can be generated by using the hands and/or upper body to strike piano keys or bow strings, among other instrumental mechanisms. The upper body can also be used to create percussive sounds by shaking a rattle or striking a drum head. Such percussive devices can be employed all throughout the body, such as in leggings attached to the lower limbs, taps placed on the heels of shoes, or simply by clapping the hands together. The use of body percussion permits acoustic rhythms to be generated through dance as much as through music-making (Brown and Parsons, 2008). The end result of this is that rhythm is a supervenient process that is found in music, dance, and in rhythmic forms of speech (such as poetic verse and group chanting). Next, drawing and pantomime are very similar systems of production. Pantomime can sometimes occur as “air drawing” – or what Ekman and Friesen (1969) refer to as “pictographic” gestures – and drawing can be thought of as iconic gesturing that leaves a trail behind in the image that forms on the canvas during the process of drawing (Yuan and Brown, 2014). Acting too can show supervenience. The dramatic character of Romeo can be portrayed by a theater actor who speaks or by a ballet dancer who is silent. In both cases, expressive gesturing with the face and body is used. The theater actor can also employ speech prosody in a way that the ballet dancer does not, while the dancer-actor can employ elaborate movement patterns (in synchronization with musical rhythms) that the theater actor does not. In addition, the dancer receives a large degree of emotional force from a musical score that is congruent with the emotional tone of the narrative.

### Combinations Between Artforms

**FIGURE 3:**
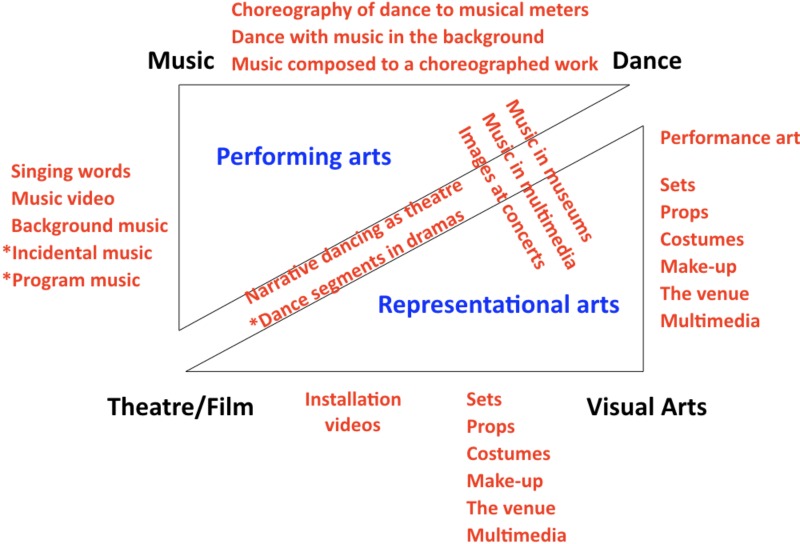
Interactions among the arts. The same double-triangle representation from Figure 1 is shown here. In red text are the six types of two-way interactions between artforms. Whereas most interactions involve simultaneous combinations of artforms, the interactions marked with asterisks (i.e., incidental music, program music, and dance in verbal dramas) are those that tend to occur in alternation with one another.

As mentioned above, the supervenience of production mechanisms creates affordances for combination, permitting artforms to couple with one another to form syntheses. The four major branches of the arts have the potential for six binary interactions (Figure 3), as described here.

(1) *Music/theater interaction*s can occur through the direct coupling between musical pitches and spoken syllables via the singing of words in operas and musicals, through the combination of music and video in music videos, through the use of background music (so-called underscore) played during the dialog and action of a film, through the “incidental music” that is performed in between the scenes of a play, or through “program music,” which refers to instrumental works composed in such a manner as to map onto the plot lines of literary or theatrical works, as is seen in Dvorak’s *Noonday Witch* or Tchaikovsky’s *Romeo and Juliet* overture.
(2) *Dance/music interactions* deal with the choreographic relationship between dance and music. This generally occurs in the two major fashions of dancing to the beat of music (as in a waltz), or dancing without a consideration for musical beats, where music mainly functions as a background element in the dance work, such as in certain forms of contemporary dance. Less commonly, music can be composed to a pre-existing dance work, as occur in some forms of contemporary dance.
(3) *Dance/theater interactions* mainly occur when dance is a type of theater work, for example in narrative forms of ballet (e.g., *Romeo and Juliet*). In musical theater works like operas and musicals, dance segments are sometimes interleaved with non-dance segments, as occurs in *West Side Story*.
(4) *Theater/visual arts interaction*. Because I conceive of the visual arts as being comprised of static objects, the major interaction between the visual arts and performing artforms such as theater is found in the static components of theastrical works, such as the sets, props, costumes, and make-up. It also covers the architectural features that make up the performance venue, including its interior and exterior design.
(5) *Dance/visual arts interaction.* Everything mentioned in the last point about sets, props, costumes, make-up, and venue applies here. A broader view of the visual arts, as developed in the 20th century, incorporates a performance component to this branch of the arts, what is referred to as “performance art” (Goldberg, 2011), which shares features with forms of contemporary dance and theater.
(6) *Music/visual arts interaction*. The interactions between the visual arts and music are the least prominent among the interactions described in this section (although see the mention of music video above). Examples include music played in exhibition spaces in museums, and images projected onto surfaces such as walls during musical concerts in concert halls. At the compositional level, there are many examples of paintings or sculptures that depict musicians and/or musical instruments, as well as musical works that were inspired by visual art works (e.g., Mussorgsky’s *Pictures at an Exhibition*).

**FIGURE 4:**
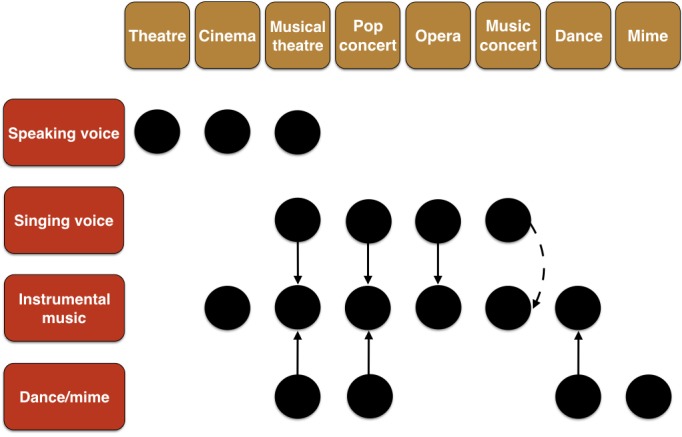
The building blocks of arts combinations in the performing arts. The figure shows eight forms of the performing arts along the top. These are then classified with respect to their inclusion of four performance modalities: speaking voice, singing voice, instrumental music, and dance (and/or mime). Black circles imply the presence of a given modality in a given artform. The vertical arrows indicate the typical directionality of coupling, suggesting that singing and dancing are very often done to the beat of instrumental music in these artforms. The dashed arrow for “music concert” implies that singing can be done either a capella or to instrumental accompaniment; likewise, instrumental music can be performed all on its own. Performance art (not shown here) sits in the categories represented by dance and mime.

As a way of demonstrating the building blocks that can be used to create artform combinations, Figure 4 shows eight forms of the performing arts along the top. These are then classified with respect to their inclusion of four performance modalities: the speaking voice, the singing voice, instrumental music, and dance (and/or mime). Based on this figure, musical theater is the closest thing to being a “total work of art” in contemporary culture (Brown and Dissanayake, 2018). It is important to note that there are combinations that are based on more than two artforms. Many pop singers dance while they sing, combining speech, music, and dance. Multimedia artforms can combine text, speech, music, still images, video, animation, and more (Costello, 2017). Having discussed the types of arts combinations that can occur, I now want to examine three issues related to such combinations: simultaneity vs. sequentiality in coupling, the strength of coupling, and the directionality of coupling.

### Simultaneity of Coupling

Whereas most interactions involve simultaneous combinations of artforms, there are other types of interactions whereby artforms occur in alternation with one another. In the case of incidental music, the music occurs between the scenes of the play, in contrast to underscore, where it occurs simultaneous with the dramatic segments of the play. In artforms such as opera, the dance segments tend to occur in alternation with the dramatic segments, rather than simultaneous with them.

### Strength of Coupling

While the discussion of combinations so far has focused on the constituent artforms that contribute to such syntheses, it is not the case that all combinations are mechanistically identical. More specifically, the *strength of coupling* between artforms can span from being very strong to being very loose. In order to exemplify this, I will examine three forms of what I will call “musical narration,” in which music is used as a means of enhancing the emotional meaning of narratives, whether they take the form of language, dance, or dramatic scenarios, as shown in Table 1. Songs with words reflect a strong coupling between music and language, wherein musical phrases and linguistic phrases are aligned at the syllabic level. Dance represents another relatively tight form of coupling, this time between body movement and musical phrases. These movements often, but by no means always, occur to the beat of the music. The coupling between dance and music tends to be looser than that between language and music, as body movement is typically only synchronized to the strongest beats in the music and not to the many levels of sub-beats present in the music. Thus, if one were to dance a waltz to the second movement of Berlioz’s *Symphonie Fantastique* (“A Ball”), one would only step to every second note of the melody, since the principal beats of the waltz rhythm are each broken down into two sub-beats in the melody. The final example of musical narration represents a loose coupling between music and narrative, as exemplified by the underscore (i.e., background music) that occurs in films. Here the music serves as a general emotive modulator of the dramatic narrative, with loose coupling occurring between the script’s sentences and the score’s musical phrases (as opposed to the musical artform of opera, where musical coupling to text is strong). The music reflects the general emotional tone of the scene, but in no way maps onto the individual words or syllables that are uttered by the actors. While multimedia is not shown in the table, musical coupling in multimedia-based forms can operate in either the manner of dance or the manner of dramatic scenarios. In situations of tighter coupling, the music may track kinetic aspects of the visuals in a direct manner (as in many children’s cartoons), whereas in situations of looser coupling, it may simply add a layer of emotional expressiveness onto the meanings conveyed by the visuals overall (as in a museum installation).

**Table 1:** Strength of coupling between artforms during “musical narration”.

	Type of interaction with music	Melodic coupling	Rhythmic coupling	Coupling
Words (texts)	Direct coupling to music	Direct coupling of syllables to pitch	Direct coupling of syllables to meters	Strong
Dance movements	Entrainment of movements to strong beats	Movements can follow melodic line	Movements coincide with strong beats	Medium
Dramatic scenarios	Music in the background	Loose coupling: emotional tone of a scene	Loose coupling: general pace of an action	Loose

*The association between music and various narrative forms is shown as a gradient of coupling strength from verbal texts to dance movements to dramatic scenarios. While multimedia is not shown in the table, musical coupling in multimedia-based forms can operate in either the manner of dance or the manner of dramatic scenarios.*

### Directionality of Coupling

**FIGURE 5:**
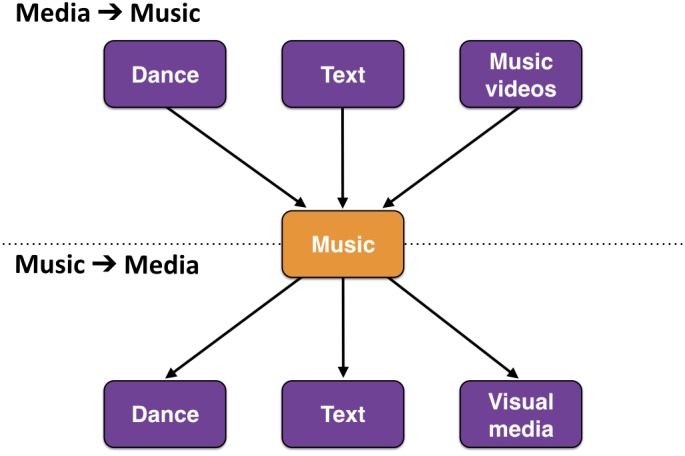
Directionality of coupling between artforms. Using music as a reference, the figure contrasts two directions of coupling: “media-to-music” and “music-to-media.” In media-to-music couplings, the music comes first, and the media attempt to conform to its structural and expressive properties. In media-to-music couplings, the media come first, and the music attempts to conform to dance movements, text, or the structure of visual media.

Combinations between artforms differ not only in the strength of their coupling but in the directionality of the coupling as well. This directionality describes which artform tends to conform to the other artform when the two come together to create a combination. In the examples presented in Figure 2, the implied directionality was that dance was choreographed to music and that music was set to the text of a poem. Figure 5 presents an analysis of directionality, using music as the reference function. The figure contrasts two directions of coupling: “media-to-music” and “music-to-media.” In media-to-music couplings, the music comes first, and the media attempt to conform to its structure. In most cases, dance is choreographed to music, although in some forms of contemporary dance a composer can compose music to a dance (shown in the lower panel). Likewise, in music videos, the song comes first, and the video elements are designed to conform with both the musical (e.g., rhythm, tempo) and narrative features of the song. In media-to-music couplings, the flow is in the reverse direction. In films, cartoons, and video games, the visual elements typically come first, and the music is added on afterwards by a composer. When it comes to creating songs with words, both directions of coupling occur quite frequently. In opera, the libretto precedes the musical score. Likewise, in standard arts songs, the text is often based on published poetry, which the composer sets to music (so-called text setting). In the reverse situation, a melody is composed first and words are added to it, such as when a piece of instrumental classical music is used as the basis for a pop song (e.g., *Full Moon and Empty Arms* based on Rachmaninoff’s second piano concerto). Songwriting teams have been known to work in both directions when coupling text and music in creating songs.

## Multisensory Perception

The counterpart to supervenient production mechanisms and artform combinations is multisensory perception, as shown at the bottom of Figure 2. Perceiving a dance-work engages systems involved in perceiving the dance movements visually and the music acoustically, while perceiving a song with words engages acoustic systems involved in both speech/language perception and music perception (including melody, harmony, and rhythm). Consider the experience of attending an opera as an audience member. This experience engages sensory and neural systems for visual perception (channels for form, color, spatial organization, motion, depth, etc.) and auditory perception (channels for speech and music). It also engages systems for action observation, social-cognition systems for the perception of body gesture and facial expression, mentalizing systems for engagement with the characters’ intentions and emotions (including potentially empathizing with the characters), and aesthetic systems for the emotional appraisal of the art work and the performance.

Now consider participating in a folk dance with multiple partners, where everyone is holding hands to form a closed circle. This activity will engage, beyond the audiovisual components present in opera viewing, additional systems for vestibular function, kinesthesis, touch, and even olfaction. Such sensory cues are not merely experiential, but are used as critical cues for entraining one’s movements with the other dancers so as to produce group coordination. For example, all dancers are attempting to entrain to the strong beats of the music that everyone is jointly hearing. The kinesthetic pushing and pulling forces that one feels from physical contact with one’s nearby neighbors can ideally reinforce the sensation of the beat coming from the music (through a temporal alignment of stress patterns). However, it can also work in conflict with it if those forces are not synchronized with the musical beat (through a misalignment of stress patterns). In the latter situation, a person experiences one rhythm in the music and different rhythm in the kinesthetic forces coming from his/her neighbors. At its best, moving in time with people by synchronizing one’s body movement with them can increase feelings of emotional attachment to them, resulting not only in an increase of social bonds but a willingness to cooperate with people and make personal sacrifices on behalf of the social group (McNeill, 1995; Reddish et al., 2013; Launay et al., 2016). The enhancement of social cooperation is perhaps the strongest functional role of the coordinative arts (Brown, 2000).

The *congruence* of sensory cues that occurs during the multisensory perception of artform combinations is a major topic of interest in the study of music in multimedia, which provides an excellent forum for examining artform combinations. Creators of multimedia works generally attempt to produce a congruence between the acoustic features of the music and the visual elements of the scene and its actions (Cohen, 2013, 2015; Eitan, 2013). For example, high pitch in music can be associated with both high spatial position and high speed in the visual domain. The same relationships obtain for loudness in the music. Interestingly, the principles for creating audiovisual congruence in multimedia artforms are the same ones as those used in dance choreography, although the congruence here is between musical features and *kinetic* features of dance movement. This establishes a consilience between multimedia and dance as multisensory artforms that are coupled with music, where multimedia emphasizes visual connections with sound, and dance emphasizes kinetic connections with sound, but where the congruences operate according to the same biological principles. From the audience’s standpoint, these are in fact identical mechanisms, since dance is perceived visually.

As a final point about the congruence of sensory cues across artforms, much research has demonstrated the similar use of cues for emotional expression between speech and music (Juslin and Laukka, 2003). Modulations of parameters such as pitch height, loudness, tempo, and articulation have been shown to have the same or similar emotional interpretations in speech prosody and music. Moreover, most of these features have parallel interpretations in dance movement as well (Sievers et al., 2013), as exemplified in the principles of Laban Movement Analysis (Newlove and Dalby, 2004). If happy speech and happy music are both associated with high pitch, loud amplitude, fast tempo, and disconnected articulation, then happy gesturing has parallel associations with high spatial positioning, large and/or forceful movements, fast tempo, and disconnected movement patterns. Hence, there is a consilience in the perception of expressive cues across acoustic, visual, and kinetic artforms (and of course their production mechanisms as well).

## Combinations of People: Coordination in Group Performance

Interpersonal coordination is one of the hallmark functions of the arts, as seen ubiquitously in the performing arts of dance and music, but also in the group chanting of text in religious and political contexts (Cummins, 2013). Group performance adds yet another layer of “coupling” to a consideration of arts combinations. At the level of the individual, it is about integrating the production mechanisms of the constituent artforms (e.g., singing words, dancing to music). At the group level, it is about maintaining that intra-individual integration while at the same time coordinating oneself with all of the other participants in the performance. Hence, a participant in a group dance not only has to dance to the beat of the music (intra-individual combination), but has to coordinate her dance movements with her partners (inter-individual combination). Interpersonal coordination in performance generally involves the playing of various coordinative roles, spanning from the stratified roles of leader and follower to the egalitarian role of co-equal.

The performing arts provide unprecedented opportunities for studying all aspects of joint action, both the production mechanisms and the perceptual cues for coordination (Sebanz et al., 2006; Keller et al., 2014; Sacheli et al., 2015). Coordination can be examined at the performer/performer level, the audience/audience level, and the performance/audience level, covering the full gamut of interpersonal interactions in public performance. Because the arts are forms of expressive behavior that function primarily in emotional communication, they also provide an excellent model for studying collective emotional expression and emotional contagion (von Scheve and Salmella, 2014). As mentioned earlier, the process of synchronizing with other people can have the effect of dissipating feelings of isolation and self-interest, and instead promoting feelings of affiliation and bonding, allowing people to abandon themselves to the group’s spirit (Durkheim, 1912/1995). The synchronous behavior that occurs in interpersonal combinations during group dancing and music-making are the closest thing that human groups ever come toward achieving the behavior of an integrated organism. In other words, the coordinative arts promote the organismal behavior (Sober and Wilson, 1998) of human groups in a manner that no other human behavior does or can.

## Sharing and Combining in the Applied Uses of the Arts

The same reasoning that has driven my discussion of the unification of the arts thus far can be brought to bear on the applied uses of the arts in therapy, education, and marketing, among many other areas. In applied uses of the arts, one or more artforms is used in order to have a positive impact on some *non-arts* behavior, such as a clinical condition, the learning of academic material in the classroom, or consumer behavior in the marketplace. Hence, the relevant interaction in this situation is not an art-art combination (as per the earlier discussions), but instead an *art-application interaction*, whatever that application may be. That said, the logic of that interaction is still based on some notion of shared production mechanisms between the artform and the application, as well as the affordances that this sharing offers for the artform to influence the application. This is typically described in the literature as a “transfer effect” (Ford and Weissbein, 1997), meaning that a process of learning or expertise in one area transfers over to improve learning and/or performance in a different area. In order for art-application interactions to be explicable, rather than mystical, it is important that there be a degree of sharing between the artform and the application at the production and/or perception levels.

Let us consider a few examples. Dance has been shown to have a positive impact on motor syndromes like Parkinson’s disease and developmental coordination disorder (Hackney and Earhart, 2010). Video games have been used to enhance visual-spatial and attentional skills (Achtman et al., 2008). Role playing methods like psychodrama are used to improve social-interaction skills (D’Amato and Dean, 1988; Kipper and Ritchie, 2003). Musical training in children is thought to have a positive impact on mathematical skills (Courey et al., 2012), especially spatial reasoning, most likely due to the experience of spatial scaling that comes from working with musical notation, not to mention the experience of fractions that occurs when interpreting the duration values of notes. Musical training also has an impact on phonological development for both first and second languages (Krizman et al., 2012), no doubt related to the pitch experience that comes from working with musical intervals, melodies, and harmonies.

Combinations of artforms provide the potential for *multi-arts applications*. For example, while there are therapies that are based on movement alone (e.g., exercising) or music alone (e.g., music therapy), dance therapy is the implementation of an arts combination (dance + music) that attempts to reap the benefits of both of the contributing artforms (Chaiklin and Wengrower, 2009). The Kodaly method of music education combines speech syllables, body movement, hand signs, and musical scales (Kodály, 1965). The same multi-arts logic can be applied to the combined effect of the music and the visuals in commercial advertising.

Most of the therapeutic examples mentioned here are based on *active* therapeutic methods, whereby a patient engages in the production of an artform. This can engage the individual alone or groups of individuals participating together. Active therapies include those that involve music-making (both singing and instrumental performance), dance/movement, writing, role playing, or painting/drawing (Gladding, 2011). This is in contrast to *passive* methods, where the patient merely perceives an artform, such as listening to music or observing a dance performance. To explain the effects of passive methods, we are less likely to invoke art-application interactions, since no production is involved. The effects might be due mainly to the aesthetic properties of the artwork and the palliative effect that this has on emotional control and coping with discomfort. If passive methods involve live performance and/or group outings, then factors related to social activity and interpersonal interaction can also be invoked.

Perhaps the most ancient application of the arts is its role in rituals as part of religious ceremonies (Durkheim, 1912/1995; Turner, 1969; Schechner, 1974, 1985; Tambiah, 1979; Dissanayake, 1988, 1992; Rappaport, 1999; Brown and Dissanayake, 2018). I argued above that ceremonial rituals were probably the first “total works of art.” Active engagement in a wide variety of artforms during religious rituals impacts non-arts behaviors related to all aspects of functioning of a social group. In general, the arts are supportive of social cooperation through their capacity for both conveying narratives and engendering interpersonal coordination. In both cases, the effects of the arts in religious rituals are of a symbolic nature, rather than being directly efficacious. There is a general agreement that the arts are a reflection of the structure and function of a society, where they reinforce social relationships and hierarchies (Lomax, 1968). In addition, they provide a forum for group assembly and thus group emotional expression. The arts serve important evolutionary functions for group survival, ultimately supporting social cooperation through the behavioral modeling of prosocial norms that occurs in narrative forms and through the symbolic sense of unity that emanates from synchronizing with group members during coordinative rituals.

## Reciprocity Between Science and Artistic Practice

An excited aspect of the science of art is not only the cross-talk among arts domains described thus far, but the reciprocal influence between scientific research and artistic practice, in other words the influence of the arts on scientific research, and the influence of science on artistic practice. While some of the scientific work on production mechanisms in the arts looks at non-artists or amateurs, others research examines experts and professionals. Thus far, the focus has been far more on performers than on creators, and thus more on performance skill than creativity. An area of interface between the two can be found in the study of improvisation, where creativity is played out during the course of performance. This has been looked at through work on piano improvisation (Limb and Braun, 2008; de Manzano and Ullén, 2012), creative drawing (Ellamil et al., 2012; Saggar et al., 2015), creative writing (Shah et al., 2013), lyrical text improvisation (Liu et al., 2012), and movement improvisation (Chauvigné et al., 2018), among other areas.

The flip side of this is the impact of scientific research on artistic practice. Composers have taken advantage of knowledge of the acoustic basis of musical intervals, harmonicity, and the operations of the cochlea. Painters have taken advantage of scientific knowledge of visual perception and color mixtures. More recently, scientific imaging techniques have had an impact on the visual arts, for example the imaging techniques that are used in molecular biology (Anker and Nelkin, 2004) and brain imaging.

## Emerging Fields in the Science of the Arts

The most established fields of research in the arts that have strong representations in both cognitive work and neuroscience are: music, aesthetics (both non-artistic and artistic), creativity (both non-artistic and artistic), and visual art (especially in connection with aesthetic studies). Fields at an intermediate level of development include dance, cinema, literature, and multimedia. Nascent fields that are in the process of establishing themselves include acting/theater, architecture, poetry, visual design, and the chemical arts. Based on the perspective of this article – with its focus on supervenience in production mechanisms and affordances for combination – the more that one understands one artform the more that one can understand about how it interacts with other artforms in production and perception. For example, “dramaturgical” approaches to social behavior argue that all human interaction is akin to a form of acting, where each person plays out different roles in different social contexts (Goffman, 1959; Shulman, 2017). To take another example, I am very drawn to the idea that we can understand architecture by using concepts from music, such as “rhythm,” where rhythm in this case refers to a recurring spatial pattern (Sterken, 2007; Chan, 2012). This would extend the concept of rhythm beyond temporal patterns in the performing arts to include spatial patterns in architecture and visual design, so that we could reasonably talk about the rhythm of a building. Goethe famously referred to architecture as “frozen music.” I think we will witness a great deal of cross-fertilization by considering how the arts relate to one another, as well as how they relate to cognition and behavior more generally.

What are the research implications of the ideas presented in this article? As mentioned earlier, the study of unification focuses on two key issues: what the arts share, and how the arts combine. Hence, I can imagine research programs based on these two issues, where the interface between them is that shared production mechanisms provide affordances for combination. Corpus analyses can investigate the manner in which creators have historically combined artforms, such as through examinations of text-setting in songs, the choreomusical relationship in dance, and set and costume design in theater. This can be done in a cross-cultural fashion to look for structural, expressive, and aesthetic universals in the arts (e.g., Brown and Jordania, 2013). Newer forms, such as multimedia, have undergone much less analysis with regards to the mechanisms by which forms combine, and so they could provide fertile ground for exploring how media artists create the particular combinations that they do. Experimentally, work on creative production in the lab could examine the manners in which creators generate combinations by manipulating the conditions by which such combinations can be formed. Likewise, perceptual studies could examine the important issue of congruence, and analyze why certain artform combinations are perceived as congruent, while others are not, as well as the impact that congruence has on people’s understanding of and liking for the artwork. Overall, a combination of corpus analyses, production studies, and perception studies could serve as the foundation for empirical research into the study of unification.

## Conclusion

This article set out to provide a manifesto for studying the arts in their collectivity, rather the traditional approach of looking at each artform independently of the others. This includes examining supervenient production mechanisms across arts domains, and how this supervenience establishes affordances for combinations between artforms. Such combinations at the production level are reflected in multisensory mechanisms at the perception level, where the goal in production is often to achieve a congruence among sensory cues such that these cues are mutually reinforcing, both sensorily and expressively. This approach toward unification serves the dual purpose of placing the arts within the context of neurocognition and placing neurocognition within the context of the arts, since the arts contain a number of art-specific modules that are not reducible to general cognitive processes. In particular, the arts provide mechanisms for interpersonal coordination and group emotional expression that have few counterparts outside of the arts. Finally, the unification of the arts proposed here is concordant with the overarching intellectual goal of creating a consilience between the humanities and the sciences.

## Author Contributions

The author confirms being the sole contributor of this work and has approved it for publication.

## Conflict of Interest Statement

The author declares that the research was conducted in the absence of any commercial or financial relationships that could be construed as a potential conflict of interest.

## Footnotes

1 I am using the term “theater” here to signify narrative artforms that are expressed through either acting or storytelling. While acting is always performative, storytelling can be done in either a performative manner (oral forms of storytelling and poetry) or in a written – hence *non-performative* – manner. This creates a complication for the scheme. Technically speaking, written literature belongs together with the visual arts, since written text is a type of image (Elkins, 1999). While I acknowledge this complication in classifying written forms of narrative in the scheme, I will not deal any further with this issue in the current article.
